# Multi-source multi-modal markers for Bayesian Networks: Application to the extremely preterm born brain

**DOI:** 10.1016/j.media.2023.103037

**Published:** 2023-12-01

**Authors:** Hassna Irzan, Michael Hütel, Helen O’Reilly, Sebastien Ourselin, Neil Marlow, Andrew Melbourne

**Affiliations:** a School of Biomedical Engineering & Imaging Sciences, King’s College London, London, SE17EU, UK; b Department of Medical Physics and Biomedical Engineering, University College London, London, WC1E6BT, UK; c Institute for Women’s Health, University College London, London, WC1E6HU, UK; d Department of Psychology, University College Dublin, Dublin, D04C1P1, Ireland

**Keywords:** Bayesian networks, Graphical models, Structure learning, Preterm birth, Brain, Neuroimaging, MRI, Diffusion MRI, fMRI, Cognitive measurements

## Abstract

The preterm phenotype results from the interplay of multiple disorders affecting the brain and cognitive outcomes. Accurately characterising these interactions can reveal prematurity markers. Bayesian Networks (BNs) are powerful tools to disentangle these relationships, as they inherently measure associations between variables while mitigating confounding factors. We present Modified PC-HC (MPC-HC), a Bayesian Network (BN) structural learning algorithm. MPC-HC employs statistical testing and search-and-score techniques to explore equivalent classes. We employ MPC-HC to estimate BNs for extremely preterm (EP) young adults and full-term controls. Using MRI measurements and cognitive performance markers, we investigate predictive relationships and mutual influences through predictions and sensitivity analysis. We assess the confidence in the estimated BN structures using bootstrapping. Furthermore, MPC-HC’s validation involves assessing its ability to recover benchmark BN structures. MPC-HC achieves an average prediction accuracy of 72.5% compared to 62.5% of PC, 64.5% of MMHC, and 71.5% of HC, while it outperforms PC, MMHC, and HC algorithms in reconstructing the true structure of benchmark BNs. The sensitivity analysis shows that MRI measurements mainly affect EP cognitive scores. Our work has two key contributions: first, the introduction and validation of a new BN structure learning method. Second, demonstrating the potential of BNs in modelling variable relationships, predicting variables of interest, modelling uncertainty, and evaluating how variables impact each other. Finally, we demonstrate this by characterising complex phenotypes, such as preterm birth, and discovering results consistent with literature findings.

## 1 Introduction

The detection of markers for certain health conditions is a challenging problem in neuroscience, emerging from the lack of sufficient data, the complex nature of the health disorder, the high number of confounding factors involved, and the capability to handle only a few features. Neuroimaging studies have used various machine-learning techniques to address these shortcomings. However, insufficient attention has been directed to Bayesian Networks (BNs) (Mateos-Pérez et al., 2018). Studies using BNs have focused narrowly on prediction or association discovery and have not fully explored the potential of BNs to investigate model uncertainty, model stability, sensitivity analysis, and markers’ joint probability density function. Furthermore, neuroimaging studies (using BNs learned from data or provided by domain experts) mostly use data from one single source, ignoring the enormous potential of merging heterogeneous data from complementary imaging modalities and cognitive assessments. The analysis of health conditions would benefit from the insights provided by integrating such a wide range of measures.

BNs are frequently used in genomics (Bielza and Larranaga, 2014), ecological and environmental analysis (Marcot, 2012), medical research (Su et al., 2013; Spyroglou et al., 2018; Seixas et al., 2014), and decision support in clinical settings (Berchialla et al., 2014). Despite being widely used in many fields, BNs are less common in neuroimaging studies. In neuroimaging, BNs have been used to achieve various ends, including discovering relationships between variables, performing classification, and probabilistic reasoning (Bielza and Larranaga, 2014). Among these, Naïve Bayes models, a simplified version of BN models, are employed in supervised classification to discriminate between mild Alzheimer’s Disease (AD) patients, subjects with mild cognitive impairment, and controls based on volumes and thickness of brain structures (Morales et al., 2013). BNs have been used with functional Magnetic Resonance Imaging (fMRI) data to estimate the interaction between the activated brain regions, as well as the direction of causal relationship (Mumford and Ramsey, 2014). Moreover, BN structure learning methods are used to investigate the association between MRI-derived measurements, genotypes, and psychometric measurements for AD individuals (Jin et al., 2016). A comprehensive discussion about BN application in neuroscience is provided in Mumford and Ramsey (2014), Bielza and Larranaga (2014).

A BN is defined by its structure and parameters. The structure is represented by a Directed Acyclic Graph (DAG), where nodes and edges denote random variables and conditional dependencies, respectively. The parameters are the conditional probabilities of the variables associated with each node (Koller and Friedman, 2009). Both the structure and the parameters are provided by domain expert knowledge, learned from data, or given by a combination of the two (Koller and Friedman, 2009). Unfortunately, expert knowledge is not available in many domains; hence, both structure and parameter learning are mainly learned from data (Koller and Friedman, 2009). Generally, BN structure learning from data has been performed following four approaches: constraint-based structure learning, score-based structure learning, hybrid algorithms, and structure learning with equivalence classes.

Constraint-based structure learning methods perform conditional statistical tests on the set of conditional independencies in the data and find a structure that satisfies these assertions (Le et al., 2016). The Peter and Clark (PC) algorithm is a well-known constraint-based algorithm (Le et al., 2016). Score-based structure learning methods consider a hypothesis space of potential structures and a scoring function that quantifies the goodness-of-fit of the structure to the data (Koller and Friedman, 2009); the Hill Climbing (HC) algorithm is an example of such a heuristic local search method. In order to find the optimal structure, the HC algorithm relies on a gradual improvement of the score function by single edge change. When no further single edge change can improve the score function, the algorithm stops (Koller and Friedman, 2009).

Hybrid algorithms combine score-based and constraint-based structure learning methods. Firstly, a skeleton (i.e., an undirected graph) is estimated based on a constraint-based algorithm. Secondly, a score-based method is applied to orient the edges. One example of hybrid methods is the Max–Min Hill-Climbing (MMHC) algorithm (Tsamardinos et al., 2006). The MMHC algorithm uses the Max–Min Parents and Children (MMPC) algorithm to find each node’s Parents and Children node sets (i.e., the skeleton) and the HC algorithm to orient the edges. The heuristic estimation of the skeleton involves conditional statistical testing in the forward and backward phases. In the forward phase, node *T* is a neighbour of the target node *N* if node *T* maximises the minimum association with node N given the powerset of the conditioning set. In the backward phase, the algorithm attempts to remove false positives by testing if nodes *T* and *N* are independent given any set of the neighbouring nodes. Another class of structure learning procedures examines the I-equivalence classes of BNs. For example, Statistically Efficient Greedy Equivalence Search (SE-GES) starts the search with an empty graph and then performs greedy edge-addition based on the best score until no further edge-addition improves the score in the forward direction. In contrast, in the backward direction, the procedure performs edge-removal until no further edge-removal improves the score (Chickering, 2020; Koller and Friedman, 2009). A more extensive discussion on structure learning algorithms is in Daly et al. (2011), Koller and Friedman (2009).

The most common BN structure learning algorithms, such as HC, PC, and MMHC, present some limitations. HC runs until no further single-edge change can improve the score function. Hence, the algorithm might stop at a local maximum or a plateau. On the other hand, the MMHC and PC algorithms inherit the limitations that characterise the statistical tests: the significance level is arbitrary and might lead to rejecting a true null hypothesis if too low or accepting a false null hypothesis if too high. Moreover, the error can grow large with multiple hypothesis testing. Furthermore, statistical tests can give reliable results when there are strong dependencies among variables, while weak dependencies might give rise to multiple errors in the final structure. While MMHC might contain the problem of false positives during the backward phase and the score-based local search, PC does not have a mechanism to cross-check the dependencies.

This paper proposes a novel hybrid structure learning approach called Modified PC-HC (MPC-HC), employing statistical testing to estimate the graph skeleton (i.e., the undirected graph) and combining equivalence search with score-based methods to orient the edges. MPC-HC searches over equivalent classes for the best scoring structures. Therefore, unlike PC, HC, and MMHC algorithms, the MPC-HC algorithm achieves the optimal solution over a larger hypothesis space by scoring over the equivalence class.

Birth before 37 weeks of gestation is known as preterm birth. This definition is further subdivided based on gestational age into very preterm birth with delivery before 32 weeks of gestation and extremely preterm birth for births before 28 weeks of gestation (Quinn et al., 2016). Preterm individuals present a broad spectrum of conditions, ranging from poor cognitive and altered behavioural profile (Agrawal et al., 2018; O’Reilly et al., 2020) to complications in various organs such as lung and brain Saigal and Doyle (2008). In addition to a high prevalence of neurodevelopmental impairments in prospective memory, visuomotor skills, and general cognitive functioning (IQ) (O’Reilly et al., 2020), studies analysing the preterm born brain have found Grey Matter (GM) and White Matter (WM) abnormalities in young adults (Irzan et al., 2021, 2020c,b,a; Jha et al., 2019) and infants (Ball et al., 2015; Batalle et al., 2017).

To achieve an integrated understanding of the effects of prematurity, a body of research has examined imaging measurements, cognitive performance, clinical measures, and their association using machine learning algorithms (Ball et al., 2015; Irzan et al., 2019, 2020c; Ball et al., 2017; Valavani et al., 2021; Ball et al., 2016; Smyser et al., 2016). A combination of forest-based feature selection technique and nonlinear support vector classifier was employed to classify a group of preterm and term-born infants based on their whole-brain functional connectivity (Ball et al., 2016). Another study applied feature selection and random forests to microstructural properties derived from diffusion-weighted MRI and clinical measurements to predict preterm infants’ language function (Valavani et al., 2021). Step-wise linear regression was used to analyse the association between the volume of cortical areas and the full-scale IQ scores (Irzan et al., 2020c). In addition, linear regression was adopted to assess the association between the cognitive scores and WM diffusivity of the thalamus and cortical areas of a group of preterm infants (Ball et al., 2015). Others used a support vector machine to distinguish preterm from control subjects based on functional resting-state data (Smyser et al., 2016). Overall, these methods are based on regression analysis and are limited because of their inability to disentangle confounding effects (King et al., 2020).

Regions of the prefrontal cortex have been associated with working memory, as fMRI studies reveal persistent activity in such areas during memory tasks (Curtis and D’Esposito, 2003). However, the prefrontal cortex is responsible for diverse executive functions, and it is associated with various cognitive functions such as visual divided attention, spatial problem-solving, and semantic processing of words (Duncan and Owen, 2000; Friedman and Robbins, 2021). Researchers drew attention to the fact that diversification of the role of the prefrontal regions might lie in its WM connectivity to other areas (Curtis and D’Esposito, 2003). For example, deep GM structures and prefrontal circuitry cooperate in multiple cognitive functions, where the deep GM structures are involved in facilitating different cognitive and executive functions (Leisman et al., 2014). The fact that preterm subjects showed abnormalities in GM and WM connectivity of deep GM and cortical regions such as the frontal lobe and sensorimotor areas (Irzan et al., 2021; Ball et al., 2016, 2015; Jha et al., 2019) raises the question of how preterm birth affects the associations mentioned above.

The body of research analysing prematurity and the relationships between poor cognitive performance and altered neuroimaging measurements (Irzan et al., 2020c; Ball et al., 2015; Irzan et al., 2019; Valavani et al., 2021; Smyser et al., 2016) has mainly focused on individual imaging modalities or single cognitive measurements. This approach might be inadequate to characterise such a complex phenotype as prematurity. Therefore, our study is motivated by the lack of existing methods to discover and model multiple heterogeneous markers of prematurity. Machine learning methods such as BNs offer a fertile ground to disentangle the interactions between the markers derived from MRI measurements and cognitive performance. Thus, we propose an efficient and versatile technique such as a BNs to model the relationship between variables from different domains, such as diffusion-weighted MRI, fMRI, and cognitive measurements of extremely preterm individuals.

We employ the novel MPC-HC algorithm to model the relationship between variables of interest. This algorithm combines constraint-based methodology, inspired by the PC algorithm, with score-based structure learning techniques influenced by the HC algorithm. The learning strategy of MPC-HC is designed to navigate through equivalent classes, primarily focusing on mitigating challenges associated with local minima and plateaus. In addition, we perform bootstrapping to investigate the stability and the confidence of the estimated BNs: for each iteration, we sample from the probability density function of the variables and estimate a BN based on that sample set. The best-scoring BN, in terms of how the data fits the structure, is used to perform predictions. Moreover, we compare the predictive power of the BN estimated by MPC-HC with that estimated by the PC, HC, and MMHC algorithms and show that the MPC-HC algorithm reconstructs the true structure of benchmark BN more accurately than the other algorithms. Furthermore, we perform sensitivity analysis to examine how the variables influence each other.

We use MPC-HC to examine the relationship between the microstructural and fMRI activation properties of brain areas and cognitive performance of a group of Extremely Preterm subjects (EP) and, for control, a group of Full-Term born subjects (FT). Specifically, we analyse the relationship between Visual Working Memory (VWM), Visual Short term Memory (VSM), Verbal Comprehension Index (VCI), Perceptual Reasoning Index (PRI), fMRI activation of the Prefrontal cortex (PRF), fMRI activation of Deep GM areas (DGM), fMRI activation of Sensory-Motor areas (MTR), the neural density of WM boundless connecting Prefrontal cortex and Sensory-Motor areas (PMN), the neural density of WM boundless linking Prefrontal cortex and Deep GM areas (PDN), and the neural density of WM connecting Sensory-Motor areas and Deep grey GM (DMN).

Overall, this work makes two contributions: first, it presents a novel BN structure learning method (MPC-HC). MPC-HC is validated by investigating its ability to retrieve the true structure of benchmark BNs. Second, the work illustrates how BN can be used to model variable associations, make predictions, model uncertainty, and assess how variables impact each other. Moreover, the work shows the potential of BNs by using the MPC-HC algorithm to analyse heterogeneous biomarkers of complex phenotypes such as preterm birth.

## 2 Methods and materials

The methods are implemented in Python programming language; we utilise the pgmpy library for BNs reconstitution (Ankan and Panda, 2015).

### 2.1 Bayesian networks: Parameter learning

A BN is defined as a DAG 𝒢 = (**X, E**) with the set of nodes **X** representing multinomial random variables **X** = {*X*_1_, …, *X*_*N*_} and the set of edges **E** expressing direct dependencies. We employ *X*_*i*_ to encode both the variable and its corresponding node. A comprehensive introduction is given by Koller and Friedman (2009).

In a Bayesian framework, performing a parameter-estimation task given a dataset 𝒟 corresponds to solving the equation:

(1) $$P(\theta |D)=\frac{P(D|\theta )P(\theta )}{P(D)}$$

where *P* (𝒟|***θ***) is the likelihood function, *P*(***θ***) is the prior over the parameters ***θ*** ∈ [0, 1], and *P* (𝒟) is the marginal likelihood of the data, this serves as a normalising constant, and it does not depend on the parameter vector ***θ***.

Considering a node of a BN representing a random variable *X*_*i*_ with *K* discrete states, the likelihood function has the form:

(2) $$P(D|\theta )=\overset{k}{\underset{K}{\prod \;}}\theta_{k}^{M[k]}$$

where ***θ***_*k*_ = *P r*(𝒟 = *x*_*k*_) and *M* [*k*] is the number of occurrences of the value *x*_*k*_ in *X*_*i*_.

A conjugate prior of the multinomial distribution is the Dirichlet distribution specified by a set of hyperparameters ⟨*α*_1_, …, *α*_*K*_ ⟩. If *P*(***θ***) is *Dirichlet(α*_1_, …, *α*_*K*_ *)*, therefore, the posterior *P*(***θ***|𝒟) is *Dirichlet(α*_1_ + *M* [1], …, *α*_*K*_ + *M* [*K*]*)* and the prediction results:

(3) $$P\left(x[M+1]=x_{k}|D\right)=\frac{M[k]+\alpha_{k}}{M+\alpha }$$

where *M* is $\sum_{k}^{K}M[k]$ and *α* is $\sum_{k}^{K}\alpha_{k}$.

### 2.2 Bayesian networks: Structure learning

The proposed methodology learns the skeleton first; then, it orients the edges.

#### Identifying the skeleton

A graph 𝒢 captures the independence in a given distribution *P*(**X**) by instantiating edges between its nodes. The skeleton of a graph is given by conditional independence tests of the form *X*_*i*_ ┴ *X*_*j*_ |**U** where the variables *X*_*i*_ and *X*_*j*_ are adjacent in 𝒢 if they cannot be separated by a conditioning set of variables **U** (Koller and Friedman, 2009). In discrete BN, chi-square (*χ*^2^) deviance statistic is used, and it is defined from the null hypothesis that *X*_*i*_ is independent of *X*_*j*_ given the conditioning set **U**. The chi-square (*χ*^2^) deviance compares the observed frequencies with the expected ones, assuming the null hypothesis is true. The expected frequencies given independence are computed as *P* (*X*_*i*_, **U**) * *P* (*X*_*j*_, **U**)*/P* (**U**) (Ankan and Panda, 2015). The algorithm starts with a fully connected graph and empty conditioning set **U**. A conditional independence test is performed in each iteration and results in either keeping the edge if the null hypothesis is rejected or removing the edge if the null hypothesis is accepted (with a threshold value of *α* = 0.01). In each iteration, a conditioning variable is added to the set **U**. The algorithm stops when the conditioning set reaches a maximum number of variables outputting a skeleton.

#### Orienting the edges

Score-based algorithms tackle BN structure learning as a model selection problem: by defining a hypothesis space of possible models, a scoring function estimates the model’s goodness-of-fit to the data to find the model with the best score. In the Bayesian framework, finding a graph that best describes the data 𝒟 corresponds to finding a graph 𝒢 that maximises the posterior probability:

(4) $$P(G|D)=\frac{P(D|G)P(G)}{P(D)}$$

where *P* (𝒟) is a normalising constant, and it is irrelevant to distinguish between graphs. *P* (𝒢) is the prior probability over the graph structures; since we do not have a specific structure-prior, this term is set to uniform. The marginal log-likelihood log*P* (𝒟|𝒢) becomes (Koller and Friedman, 2009):

(5) $$score_{BIC}(G\;:\;D)=\ell \left({\hat{\theta }}_{G}\;:\;D\right)-\frac{\log M}{2}\text{Dim}[G]$$

where $\ell \left({\hat{\theta }}_{G}\;:\;D\right)$ is the likelihood score, *M* the number of training instances, and Dim[𝒢] is the number of independent parameters in the network 𝒢. The likelihood term favours models with a better fit, while the penalising term tries to keep the number of independent parameters, and hence the graph complexity, down.

Assuming that the data 𝒟 is sampled from an underlying distribution P* (**X**), which is induced by a BN 𝒢* over **X**, the goal is to recover 𝒢*. However, 𝒢* is not uniquely identifiable from the data 𝒟 as all the BNs in the same I-equivalence class of 𝒢* are an equally good fit for P* (**X**), and hence have the same *score*_*BIC*_ (Koller and Friedman, 2009). Two graphs 𝒢_1_ and 𝒢_2_ are I-equivalent if they have the same skeleton and same set of immoralities. An immorality is a V-structure of the form *X*_*i*_ → *X*_*k*_ ← *X*_*j*_ with no edge between *X*_*i*_ and *X*_*j*_ (Koller and Friedman, 2009). The consistency of *score*_*BIC*_ implies that the graph 𝒢*, or any DAG that is its I-equivalent, maximises the score, while non I-equivalent structures have strictly lower score (Koller and Friedman, 2009). Thus, the best we can do is to recover a BN that belongs to the same I-equivalence of 𝒢*.

**Fig. 1:**
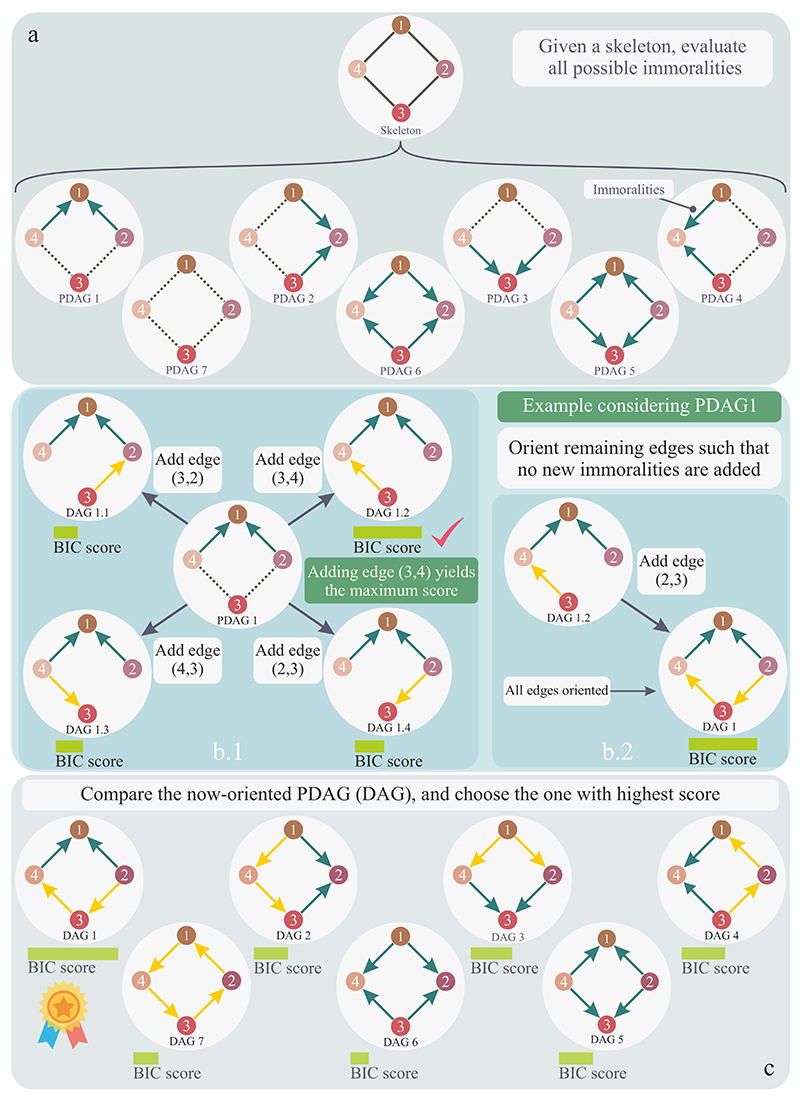
Main steps of structure learning. (a) Once the skeleton is estimated, we compute all the Partially Directed Acyclic Graphs (PDAG) by evaluating all the possible immoralities’ configurations. (b) The remaining edges of the PDAG are oriented such that the BIC score is maximised, and no other immoralities are added to the structure. (c) Once all the PDAGs are oriented to obtain Directed Acyclic Graphs (DAG), the one with the maximum BIC score is chosen.

Taking advantage of the I-equivalence class, one can evaluate one DAG per I-equivalence class over the set of possible solutions and consider the one that scored the highest. Therefore, we propose evaluating all the partially directed acyclic graphs (PDAG) identified by all the possible immoralities’ configurations in the skeleton. The process starts by considering one possible configuration of immoralities and orienting the remaining edges with specific rules. In addition to graph acyclicity, the legal operators that connect different solutions are edge orientation, edge deletion, and edge reversal of the edges already in the skeleton. Importantly, no additional edges are permitted, and no changes to immoralities should occur during this process. The algorithm performs the legal operator with the highest score. The edges in the immoralities set can neither be removed nor flipped (fixed edges). Once a legal operator is performed, the algorithm evaluates the *score*_*BIC*_ of that structure. The process iterates until no further edge adjustment improves the *score*_*BIC*_ outputting a DAG structure 𝒢. Fig. 1 outlines the main steps of the structure learning process described above.

### 2.3 Data

A dataset of psychological and neuroimaging measurements from 138 subjects aged 19 years was collected by the EPICure project, a population-based study of survival and long-term health status in extremely premature infants in the United Kingdom and the Republic of Ireland (EPICure@19; www.epicure.ac.uk) The dataset consists of a group of 85 EP and a group of 53 FT, matched by social status and economic background (O’Reilly et al., 2020). Ethical approval was granted by the South Central – Hampshire A Research Ethics Committee (reference 13/ SC/0514). All participants gave informed consent before taking part in the experiment. We extract the following markers from the EPICure dataset.

#### Psychological markers

The cognitive functions of the subjects are evaluated using *Wechsler Abbreviated Scale of Intelligence* (WASI-II), from which Verbal Comprehension Index (VCI) and Perceptual Reasoning Index (PRI) standardised scores are derived. In addition, Visual Working Memory (VWM) and Visual Short-term Memory (VSM) scores are assessed using *Automated Working Memory Assessment* (Alloway, 2007). A detailed description of how these tests are conducted is in O’Reilly et al. (2020).

#### Neuroimaging markers

Based on Geodesic Information Flow (GIF) (version 2), tissue parcellations of the corrected T1-weighted volumes are obtained, and a total of 121 Regions Of Interest (ROIs) are determined (Cardoso et al., 2015). A microstructural network is defined by a graph 𝒮_*n*_ = (**V**_**s**_, **E**_**s**_) with **V**_**s**_ the set of nodes given by the unique ROIs and **E**_**s**_ the set of edges connecting **V**_**s**_. Each edge (*i, j*) links two ROIs *i* and *j* with a connection strength defined as the mean neural density of the WM bundles connecting the two ROIs *i* and *j*. Maps of neural density are estimated by fitting the diffusion-weighted data to the NODDI model (Zhang et al., 2012). A functional network is defined by a graph ℱ_*n*_ = (**V**_**f**_, **E**_**f**_) with **V**_**f**_ the set of nodes given by the individual ROIs and **E**_**s**_ the set of edges linking **V**_**f**_. Each edge (*i, j*) linking two ROIs *i* and *j* has a connection strength equal to the pair-wise partial correlation between the time courses of the ROIs *i* and *j*. The microstructural and functional networks, 𝒮_*n*_ and ℱ_*n*_, are computed for each subject. The details of the acquisition protocols, specifications, and pre-processing steps of the MRI data are reported in previous works (Hütel, 2021; Irzan et al., 2021).

From ℱ_*n*_, we evaluate the total weight of the set of edges within ROIs of the Prefrontal cortex (PRF), within Sensory-motor ROIs (SMA), and within Deep GM ROIs (DGM). These ROIs are defined from merging more brain regions of the GIF parcellations (Cardoso et al., 2015). Section 1 of the Supplementary Material (SM) reports the details on how these ROIs are defined. From 𝒮_*n*_, we consider the mean weight of the set of edges connecting PRF and SMA (PSN), PRF and DGM (PDN), and DGM and SMA (DSN).

### 2.4 Experiments

#### 2.4.1 Data augmentation and discretisation

Bootstrapping and training a BN need a high number of data samples. Kernel Density Estimation (KDE) estimates the probability density function of the variables in the dataset (*PDF*_*KDE*_). We fix the kernel to be Gaussian while we use likelihood cross-validation to estimate the best bandwidth (Qi Li, 2006).

One significant disadvantage of using BNs is that their software implementation does not support continuous variables. Thus, although discretisation entails loss of information, it is necessary for BN development. For the analysis, we partition the data into two states using the KMeans clustering procedure. In addition, we split the data into an 80% training set and a 20% testing set. We apply data augmentation and discretisation to the training and testing sets separately. The training set is used to estimate the parameters and the structure of the BNs, while the testing set is employed to assess the prediction power of the estimated BNs.

#### 2.4.2 Prediction and performance evaluation

We use the BN that has the highest *score*_*BIC*_ over bootstrapping to make predictions on the testing data. The inputs to the model are all variables except the variable to be predicted. The model outputs the posterior probability of each state *k*. We convert probabilities into states with a decision threshold of 0.5. We evaluate the performance and the uncertainty of the estimated BN by computing the accuracy and assessing the uncertainty of the posterior probability. Specifically, we use the posterior probability certainty index (PPCI) (Marcot, 2012):

$$\text{PPCI}=100(1+\frac{\sum_{k=1}^{K}p_{k}L}{\ln(K)}),\;\;\;\text{where}\;\;L=\{\begin{array}{c} \ln(p_{k})\;p_{k}>0\\ 0\;p_{k}=0 \end{array}$$

where *K* is the number of states, and *p*_*k*_ is the posterior probability of state *k*. PPCI ranges between a minimum of zero and a maximum of 100. Higher values indicate that one or few states have a higher probability and, therefore, greater certainty. Considering that PPCI is computed for each sample, we report its arithmetic mean for clarity purposes.

The predictions’ accuracy is computed as the ratio between the correctly predicted samples over all the samples.

#### 2.4.3 Validation of the MPC-HC algorithm

We investigate the quality of the BNs estimated by the MPC-HC algorithm by comparing their predictive power to that of BNs estimated by other structure learning algorithms. Specifically, we perform BN structure learning using the MPC-HC, PC, HC, and MMHC algorithms and make predictions with the estimated structures. We compare the prediction performance of the estimated structures obtained. We use the EPICure dataset to compare the prediction performance.

Moreover, we evaluate the performance of MPC-HC, PC, HC, and MMHC algorithms in accurately retrieving the true structures of benchmark BNs, including the Cancer network, Asia network, Alarm network, Survey network, and Sachs network (Marco, 2021). This evaluation is conducted using quantitative metrics of F1 and Structural Hamming Distance (SHD) scores. Specifically, we sample 3000 samples from each benchmark BN and perform structure learning using MPC-HC, PC, HC, and MMHC algorithms. For HC, MMHC, and MPC-HC, the scoring function is *score*_*BIC*_, while the statistical threshold is *α* = 0.01 for PC, MMHC, and MPC-HC.

#### 2.4.4 Bootstrapping: analysis of confidence

We assess the confidence and stability of the learned DAGs with non-parametric bootstrapping (Friedman et al., 1999). The procedure starts by sampling the dataset *D*_*i*_ from the probability distribution *PDF*_*KDE*_, which is estimated from fitting the original data to KDE (Parzen, 1962). A DAG structure 𝒢 is estimated based on the dataset *D*_*i*_ as described in Sections 2.1 and 2.2. This procedure is repeated *m* times and the quantity *P*_*conf*_ is defined as (Friedman et al., 1999):

(b) $$P_{conf\;}=\frac{100}{m}\overset{m}{\underset{i=1}{\sum }}1\{e\in G(D_{i})\}$$

where 1{*e* ∈ 𝒢(*D*_*i*_)} is one if the edge *e* is present in 𝒢 and zero otherwise. The quantity *P*_*conf*_ quantifies the confidence that the edge *e* ∈ 𝒢*. *P*_*conf*_ ranges between zero, if *e* never appears in 𝒢, and 100 if *e* is estimated *m* times.

#### 2.4.5 Sensitivity analysis

Sensitivity analysis measures how evidence about one variable *F* may influence the belief in a target variable *Q*, specifically its posterior probabilities. This analysis can be performed by measuring the mutual information *I*(*Q, F*) (Pearl, 1988), calculated as the expected reduction in entropy *H* (*Q*) given new evidence *F* such that:

$$I(Q,F)=100\cdot \frac{H(Q)-H(Q|F)}{H(Q)}$$

The entropy reduction *I* has a minimum value of zero when variables *Q* and *F* are independent and a maximum value of 100 when *P* (*Q*|*F*) is either zero or one (certainty about the outcome of *Q*, given the evidence *F*).

The aim is to quantify how new evidence *F* reduces the target variable’s uncertainty by driving its posterior probabilities close to zero and one. Small values of *I*(*Q, F*) indicate that knowing *F* minimally reduces the uncertainty about *Q*, and higher values of *I*(*Q, F*) indicate that *F* greatly explains *Q*, and vice-versa.

## 3 Results

### 3.1 Data discretisation

The discretisation process has two states for each node (variable): low and high. The cut-off values for data discretisation are presented in Table 1.

### 3.2 Validation of the MPC-HC algorithm

**Fig. 2:**
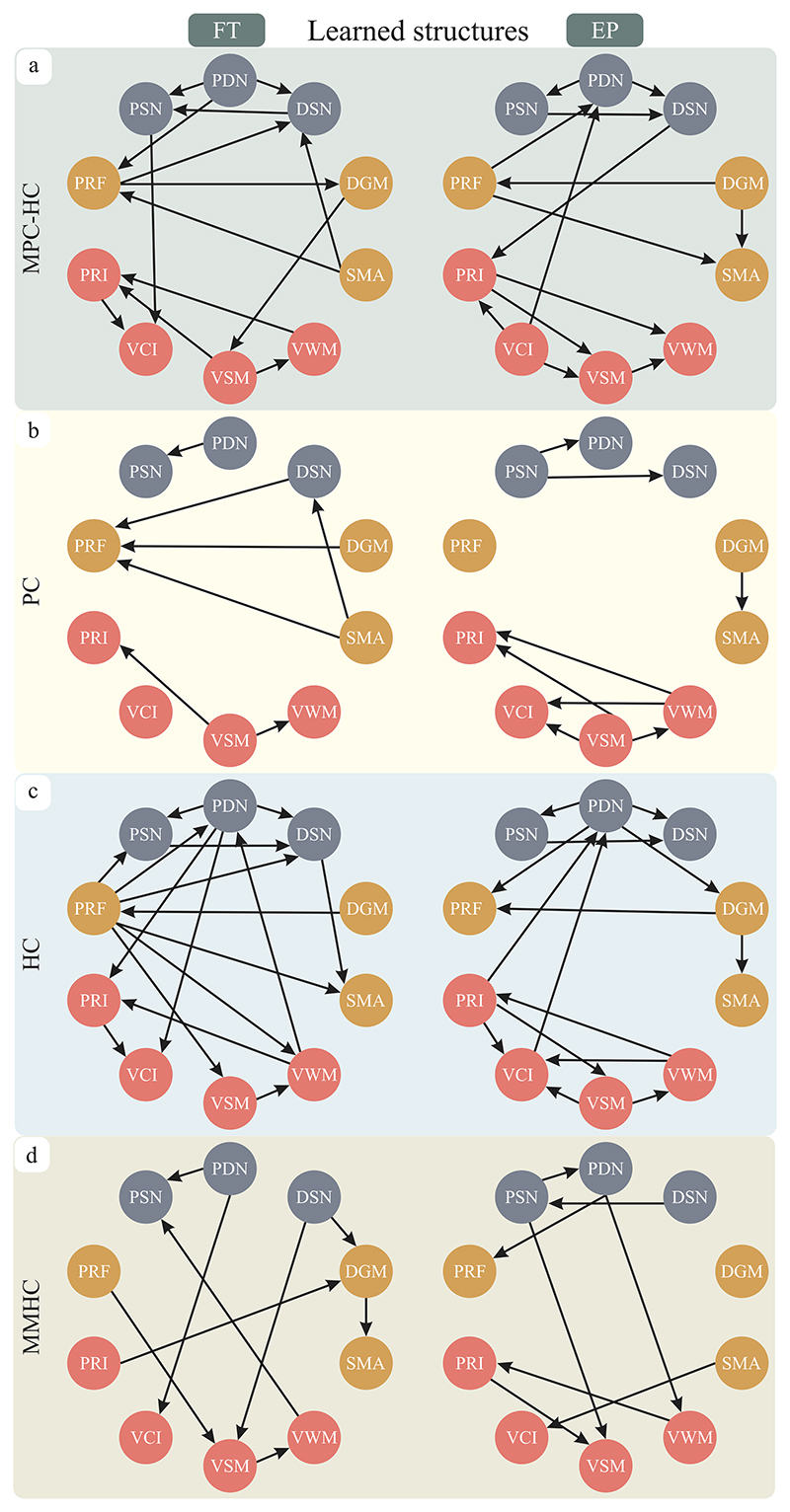
Bayesian networks discovered for Full-Term (FT) and Extremely Preterm born subjects (EP) by applying (a) MPC-HC, (b) PC, (c) HC, and (d) MMHC algorithms. The nodes in red indicate the psychological variables, while the grey and yellow nodes refer to the neural density of white matter and fMRI variables, respectively.

We perform *m* = 1000 bootstrapping iterations; over each iteration, we sample 3000 samples from *PDF*_*KDE*_ and estimate a BN structure using MPC-HC algorithm. Fig. 2a shows the structures that achieved the highest *score*_*BIC*_ for EP and FT individuals. In addition, the BN structures estimated by the PC, HC, and MMHC algorithms are shown in Figs. 2b, 2c, 2d, respectively. Overall, there are distinct patterns in edge instantiation among the PC, HC, MMHC, and MPC-HC algorithms. Specifically, the PC and MMHC algorithms exhibit a conservative approach, resulting in the absence of connections for certain nodes (PRF and VCI for PC and DGM for MMHC). In contrast, the HC algorithm instantiates many more edges, and MPC-HC instantiates fewer edges than HC and more than PC and MMHC. Importantly, all the MPC-HC, PC, and HC algorithms consistently estimated the edges PDN-PSN, DSN-SMA, VSM-VWM, and DGM-PRF for FT subjects; similarly, the edges PRI-VSM, DGM-SMA, PSN-DSN, PSN-PDN are consistent for EP subjects. Moreover, the structures estimated by MMHC are less consistent than other algorithms.

**Fig. 3:**
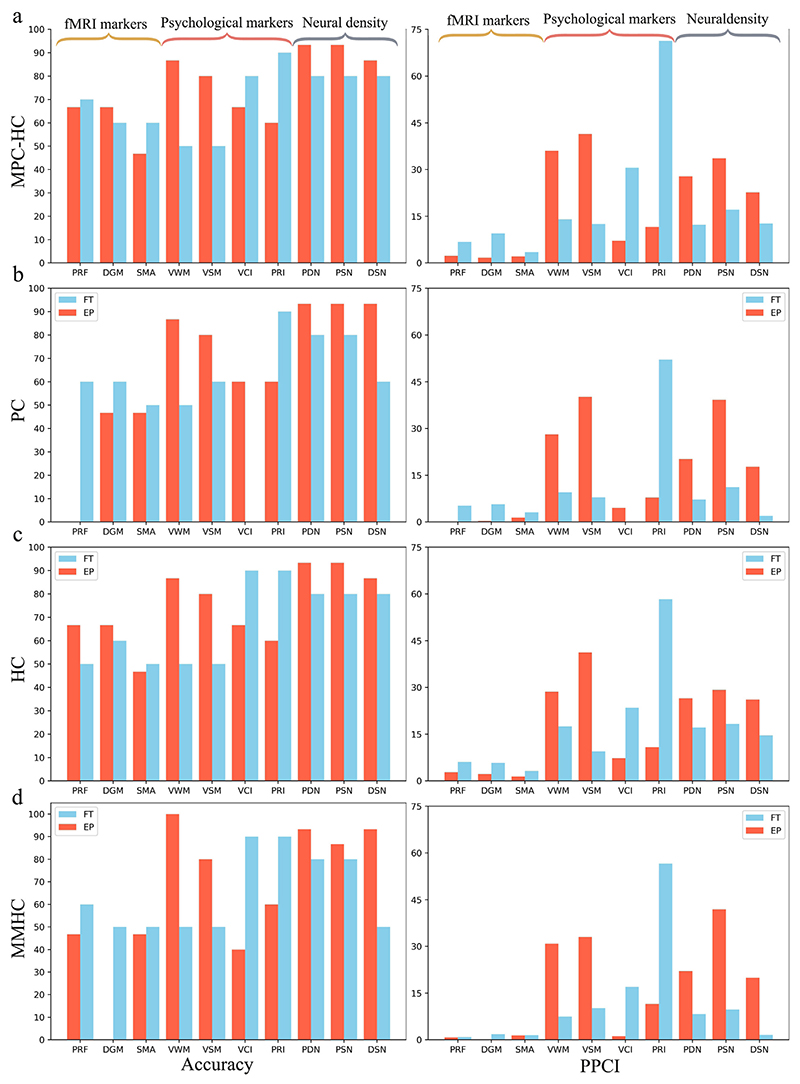
The predictive power of the BNs estimated by (a) MPC-HC, (b) PC, (c) HC, and (d) MMHC algorithms, respectively. Left: prediction power of Extremely Preterm (EP) and Full-Term born subjects (FT) BNs as measured by the accuracy of predicting states of the variable (*x*-*axis*). Right: evaluation of the Posterior Probability Certainty Index (PPCI) for EP and FT BNs. The node PRF is not connected in the BN estimated by PC for the EP group; hence, the accuracy and PPCI bars are absent, and the same for VCI for FT BN. Similarly, the node DGM is not connected in the BN estimated by MMHC for EP subjects; therefore, the DGM bar is absent for EP BN.

The predictive power of the EP and FT BNs estimated using MPC-HC, PC, HC, and MMHC algorithms is summarised by Fig. 3. Overall, the algorithms better predict the neural density measurements that show the highest accuracy scores, while the accuracy scores of the psychological variables are slightly higher than that of the fMRI variable. The PPCI scores reflect the accuracy outcome, i.e., the algorithms are more confident in predicting the neural density and the psychological variables. In contrast, fMRI variables have lower PPCI across the four algorithms and the EP and FT groups, reflecting less predictive certainty. The average accuracy of the PC algorithm (*acc*_*EP*_ = 66%, *acc*_*FT*_ = 59%) and the MMHC algorithm (*acc*_*EP*_ = 64%, *acc*_*FT*_ = 65%) is impacted because some nodes are not connected. The average accuracy score for MPC-HC (*acc*_*EP*_ = 75%, *acc*_*FT*_ = 70%) is slightly higher than that of HC (*acc*_*EP*_ = 75%, *acc*_*FT*_ = 68%). However, except for the PC algorithm, the Mann–Whitney U test indicates that there is no statistically significant difference in the performance of the models, including both accuracy and PPCI. This observation may be attributed to the limited sample size.

**Fig. 4:**
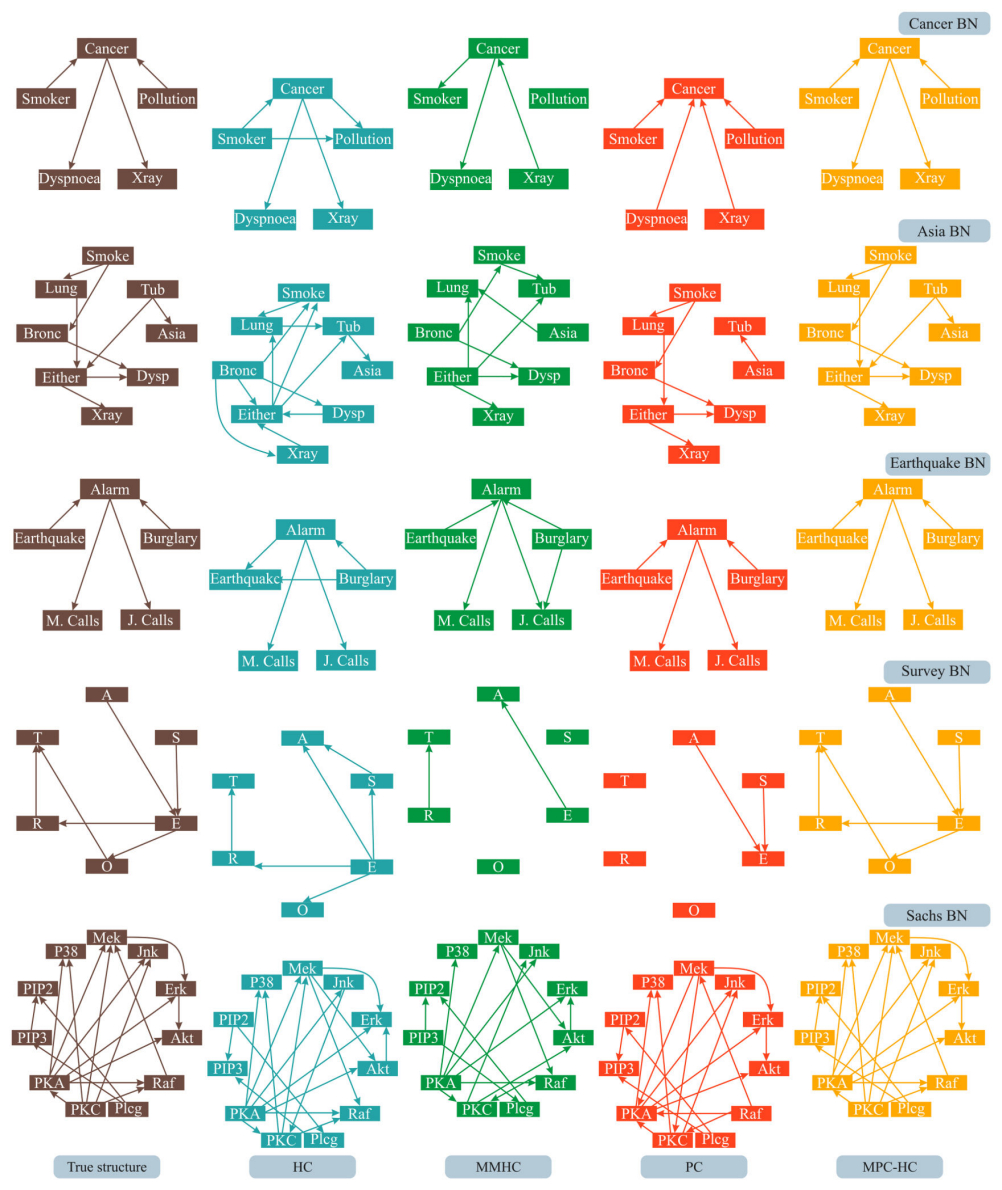
Structure estimates of benchmark Bayesian Network (BN) as computed by Hill Climbing (HC), Max–Min Hill Climb (MMHC), Peter and Clark (PC), and Modified PC-HC (MPC-HC) algorithms. On the left, the true structure of the BN is also displayed (brown).

**Fig. 5:**
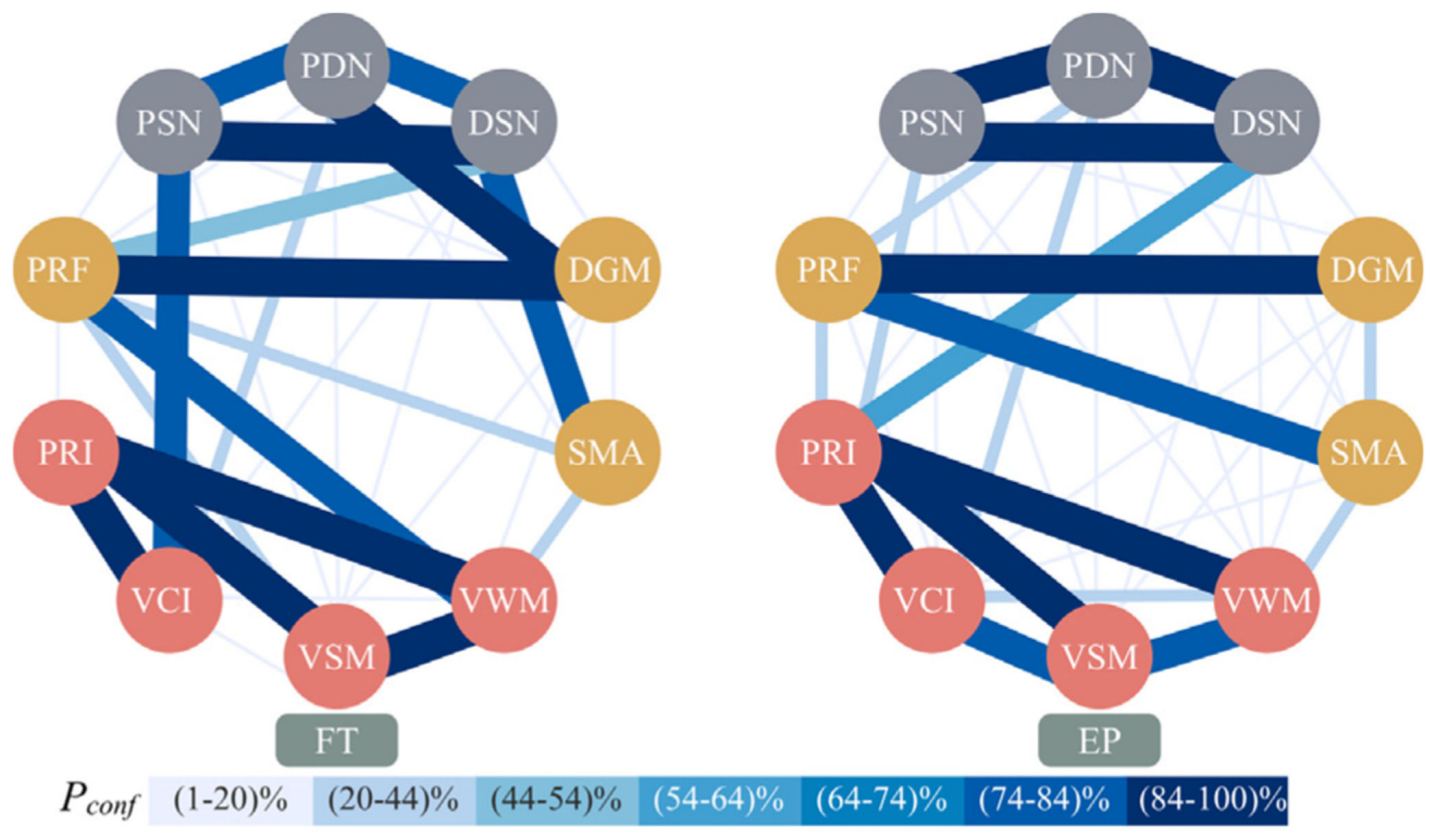
The bootstrapping results for Full-Term (FT) and Extremely Preterm born subjects (EP) BNs show the frequency *P*_*conf*_ with which each edge has been identified during structure learning with MPC-HC algorithm. When the frequency of an edge is high, there is higher confidence that the edge is real. The frequency of the edge is directly proportional to its thickness and colour: the higher the frequency, the thicker and darker the edge.

The results of structure learning of benchmark BNs using the HC, MMHC PC, and MPC-HC algorithms are shown in Fig. 4. The Figure shows that the MPC-HC algorithm retrieves the true structures of all benchmark BNs, while HC favours instantiating more edges, and MMHC and PC tend to instantiate fewer edges. The quantitative evaluation of these models through F1 and SHD scores (refer to Figure 5 in SM) further highlights this. In particular, MPC-HC consistently achieved the highest scores for both F1 and SHD across all BN benchmarks. Following, PC achieved the second-best score for almost all the benchmarks, while HC and MMHC exhibited comparatively lower performance.

### 3.3 Bootstrapping: analysis of confidence

The frequency *P*_*conf*_ by which each edge is estimated over the bootstrapping process is summarised by Fig. 5. The edges PRI-VCI, PRI-VSM, PRI-VWM, PRF-DGM, and PSN-DSN have *P*_*conf*_ ≥ 84% for both EP and FT BNs. In addition, the edges VSM-VWM, and PDN-DGM have *P*_*conf*_ ≥ 84% for FT BN only, while the edge PSN-PDN has *P*_*conf*_ ≥ 84% for EP BN only. For the EP BN, 60% of the edges have *P*_*conf*_ ≤ 10%, and only 16% have *P*_*conf*_ ≥ 87%. For FT BN, 49% of edges have *P*_*conf*_ ≤ 10%, and 20% have *P*_*conf*_ ≥ 87%. Hence, overall, EP BN presents more noisy edges. In addition, Fig. 5 shows that there is more confidence in the edges within the same source variables for both groups: i.e., within psychological variables and within imaging variables.

### 3.4 Sensitivity analysis

**Fig. 6:**
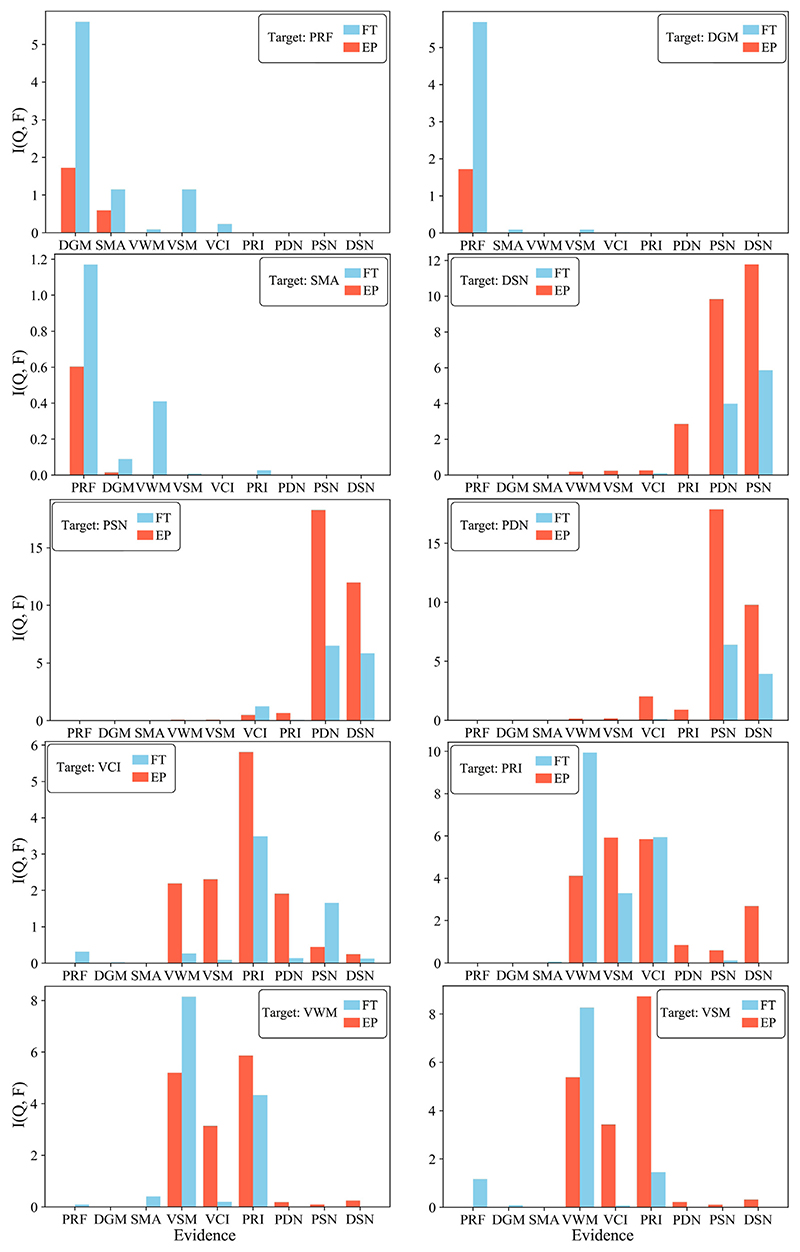
Sensitivity analysis examining the impact the variables have on each other for Extremely Preterm (EP) and Full-Term born subjects (FT) BNs. The bar plots show the percentage of reduced entropy the target variable has when the evidence variables are given. The evidence variables are shown along the *x*-*axis*.

The BN structures estimated by MPC-HC (top of Fig. 2) are used to perform sensitivity analysis, the results of which are shown in Fig. 6. Overall, the influence between variables generated from the same data source is more substantial than that generated from different data sources. For example, PDN and DSN have a greater influence on PSN than any fMRI or psychological variables. The entropy reduction is the lowest for the fMRI variables, particularly SMA is minimally influenced by PRF (1.18%), DGM (0.18%), and VWM (0.4%). On the other hand, the entropy reduction of the variables based on neural density is the highest, followed by the psychological variables. For both groups, the pairs DGM-PRF, VCI-PRI, VWM-VSM, and the triple PSN-PDN-DSN greatly influence each other. This result means that if the chance of having high VWM is 50% for FT, this probability will reach 70% once we have new evidence that VSM is also high. Similarly, the probability of low neural density in PSN is 72% for EP; such probability increases to 91%% if new evidence of low neural density of DSN is given.

Fig. 6 shows that individually, the imaging markers have little impact on the psychological variables and vice-versa. However, when considering combined imaging markers as evidence variables, the mutual influence between the psychological and the imaging variables is more substantial in EP than in FT. Specifically, the probability of EP having low PRI goes from 55% to 63% when evidence of low SMA, PRF, DGM, PDN, DSN, and PSN is given; it remains minimally changed for FT. Similarly, the probability of low VCI increases from 47% to 58% when SMA, PRF, DGM, PDN, DSN, and PSN are also low, while it barely increases for FT (from 20% to 22%). Furthermore, the probability of low VSM rises from 76% to 82% once SMA, PRF, DGM, PDN, DSN, and PSN are also low, while it remains almost the same for FT (48% vs 49%). Likewise, the probability of low VWM increases from 85% to 87% when low SMA, PRF, DGM, PDN, DSN, and PSN values are given with the probability of FT changing minimally (from 50% to 51%).

## 4 Discussion

In this work, we present a novel Bayesian Network structure learning algorithm called Modified PC-HC (MPC-HC). The algorithm combines concepts of statistical testing and search-and-score approaches. MPC-HC first reconstructs the skeleton of a Bayesian Network by testing for conditional independence; then, it evaluates all possible partially oriented graphs given by the immoralities (Fig. 1). For each immorality configuration, the algorithm orients the edges of the skeleton by maximising the *score*_*BIC*_ function. Edge orientation follows the specific rules of acyclic graphs, with no addition of edges not in the skeleton or edges that would alter the immoralities set. Once all the configurations are assessed, the BN with the highest *score*_*BIC*_ is chosen. The results show that MPC-HC can reconstruct the benchmark Bayesian Networks’ true structure (Fig. 4) while achieving higher accuracy scores (Fig. 3). In addition, combined with bootstrapping, MPC-HC estimates the confidence in instantiating edges (Fig. 5).

Using MPC-HC, we analyse a dataset of extremely preterm young adults by selecting markers from their psychological evaluation and neuroimaging measurements. The intent is to reveal the relationship between these markers and investigate the long-term changes due to extreme preterm birth compared to term birth (Fig. 2). As the ground-truth BN structures are not available for such data, we study the stability of the learned BNs and quantify the confidence of each edge by using non-parametric bootstrapping (Fig. 5). We also demonstrate the predictive power of the estimated BNs and investigate the impact the variables have on each other by performing sensitivity analysis (Fig. 6).

The analysis of the predictive power of the EPICure BNs shows that the markers based on neural density have the highest accuracy and PPCI for both EP and FT and across the BN learning algorithms, followed by the psychological markers and fMRI markers (Fig. 3). Furthermore, the correlation between the markers of neural density and between the markers of psychological measurements is higher than between the fMRI markers. This suggests that these markers are more predictive of one another. The sensitivity analysis supports this conclusion: the neural density markers extensively explain each other, and similarly, the psychological markers explain each other. This effect is not seen in the fMRI markers. In terms of the accuracy achieved and certainty about the predictions (PPCI), the performance of MPC-HC is slightly higher than that of HC (Figs. 3a and 3c); the performance of the BNs estimated by the PC and MMHC algorithms (Figs. 3b and 3d) is the lowest. This may be due to a less complex BN structure. While the performance of MPC-HC does not exhibit a significant statistical difference compared to the HC and MMHC models, it is important to consider that the statistical comparison may be constrained by the relatively small sample size, comprising only ten data points.

The sensitivity analysis suggests that same-source markers influence each other more substantially than different-source variables. For example, the psychological markers influence each other more than the imaging markers and vice-versa. This result is coherent with the fact that one performs poorly in more than one cognitive task, while the association between imaging and psychological markers is more complex, generally weaker, and subject to multiple confounding. Moreover, when considering many imaging markers as evidence to the reduction of entropy of a target psychological marker, the results suggest greater entropy reduction, with more substantial effects in EP subjects. The implications of such a result are twofold. Firstly, low neural density and low fMRI connectivity affect mainly EP’s cognitive scores, which is in line with the prematurity literature highlighting how the neuroimaging measurements are associated with low cognitive outcome (Ball et al., 2015; Irzan et al., 2019, 2020c; Ball et al., 2017, 2016). Secondly, preterm birth has a broad and complex effect on the brain; such an effect is better captured by considering multiple markers.

The bootstrapping results show high confidence in the edges between neural density markers and between psychological markers. This finding is consistent with the sensitivity analysis results and further demonstrates that the neural density markers depend greatly on each other, and the same for the psychological markers. The high confidence in the edges between psychological markers suggests that these links are reliable; such relationships are consistent with the literature. For example, the edge VCI-PRI is consistent with knowing that verbal and perceptual abilities correlate. Moreover, VWM requires elements of VSM, hence the link VSM-VWM.

Bootstrapping analysis shows differences between EP and FT BNs. For example, the results suggest high confidence in the edge between the neural density of the WM between the prefrontal and somatosensory cortex (PSN) and verbal comprehension (VCI) in FT, while such a link has low confidence in EP. Research suggests that WM circuits such as the arcuate fasciculus and the superior longitudinal fasciculus are involved in language functions (Friederici and Gierhan, 2013). Specifically, these WM circuits connect the inferior frontal lobe, temporal lobe, and somatosensory areas. Each of these areas is involved in a specific language processing task (Friederici and Gierhan, 2013). It is possible that the WM circuit that we defined connecting the prefrontal and somatosensory cortex (PSN) is a subset of the WM circuits involved in the language function. Furthermore, there is high confidence in the edge drawing direct dependency between areas of the prefrontal cortex (PRF) and visual working memory (VWM) in FT BN; this is consistent with the knowledge that frontal regions are implicated in working memory performance as observed by fMRI studies (Curtis and D’Esposito, 2003). The high confidence in the association between fMRI and neural density markers, namely PDN-DGM and DSN-SMA, is found only in the FT group. The association between functional and diffusion measurements might reflect the common understanding that functional and diffusion properties are associated (Deligianni et al., 2016). Overall, the changes between FT and EP BNs might indicate alterations owing to prematurity, and future studies should address such changes.

In terms of quality of structure reconstruction, MPC-HC outperforms the PC, HC, and MMHC algorithms; this is shown in Figs. 4 illustrating the reconstructed structures of benchmark BNs and Figure 5 of the SM depicting the F1 and SHD scores. While the quantitative scores suggest that MPC-HC consistently retrieved the true structure, the BNs estimated by the MMHC and PC algorithms generally have fewer edges than those estimated by HC and MPC-HC algorithms. Given that the significance threshold (*α* = 0.01) for the statistical tests is the same for the PC and MPC-HC algorithms, the complexity of the skeleton must be similar. Therefore, the edges are removed during edge orientation in the PC algorithm. Conversely, BNs estimated by HC present more edges. Hence, it is possible that the *score*_*BIC*_ might favour more complex models, as the sample size (*M* = 3000) has a stronger effect on the likelihood function term than on the regularisation term. The paucity of edges in the BN structures estimated by MMHC might be due to the skeleton estimation step. Specifically, the backward steps of the algorithm might have removed many edges.

The fact that the MPC-HC algorithm estimated the true BN structures suggests that combining statistical testing, search-and-scoring, and class equivalence can avoid the pitfalls of every single method and achieve the global maximum empirically. In fact, achieving a global maximum is theoretically guaranteed if the statistical tests can identify true dependencies (true skeleton). While such a requirement is valid for the datasets we sampled from the benchmark BNs, it is hard to guarantee for real-world and noisy data like the young adult cohort we presented. In such datasets, spurious statistical dependencies might occur in the estimation of the skeleton; however, the spurious edges might be filtered out by the edge orienting stage of the MPC-HC algorithm as described in Section 2 of the SM. Such a result follows from the fact that MPC-HC searches over class equivalence; hence it has access to the entire hypothesis space given the skeleton. Access to a larger hypothesis space is the main difference between the MPC-HC algorithm and the PC, HC, and MMHC algorithms. Such a characteristic allows the MPC-HC algorithm to reach the optimal solution after searching all the hypothesis space given the skeleton.

The number of single immoralities defines the complexity of the algorithm. Therefore, it is hard to establish a precise association between the number of graph nodes and the size of the search space. However, given a set of single v-structures of size *r* in the skeleton, the MPC-HC algorithm needs to search 2^*r*^ graphs as a worst-case scenario. Furthermore, because some immoralities cannot be combined as they introduce cycles in the graph, 2^*r*^ is an upper bound for the worst-case scenario. Further details about the computational complexity of MPC-HC are described in Section 4 of the SM.

In the MPC-HC algorithm, the ability to instantiate an edge is established by the statistical tests and the Bayesian score. Therefore, the sample size affects the MPC-HC algorithm similarly to the statistical testing and the Bayesian score. In the skeleton estimation step, the MPC-HC algorithm might instantiate more edges in large datasets and fewer edges in small datasets. The Bayesian score orients the edges, and it is composed of two terms: the likelihood term and the penalising term. For the likelihood function, the size of the training set affects the stability of the maximum likelihood estimate: the estimates are more stable with larger datasets. To summarise, for small datasets, the edges are fewer because of the statistical testing step, might be affected by noise in the data, and therefore are more variable. However, the edges are more consistent for larger datasets, and the Bayesian score will tend to reduce them. Section 3 provides in-depth insights into how the MPC-HC algorithm’s effectiveness in learning BN structures is influenced by changes in sample size.

Furthermore, it is worth noting that the choice of statistical significance is somewhat arbitrary, often set at a threshold of *α* = 0.01. Such a threshold can potentially be too high, introducing spurious edges, or too low, overlooking true dependencies. Nevertheless, our empirical findings demonstrate that the edge-orienting step effectively eliminates spurious edges from the skeleton, as these edges tend to have lower *score*_*BIC*_. These results are presented in Section 2 of the SM. Therefore, the MPC-HC method might be challenged when the statistical threshold is too strict with respect to the independence tests; however, with higher statistical thresholds, the MPC-HC algorithm retrieves the true structures by removing spurious edges.

As part of the BN estimation process, individual markers are discretised into two states, namely high and low; although this may simplify the categories of complex results, it is not a limitation of the BNs as one can discretise the variables into more numerous states. In addition, more sophisticated discretisation techniques such as dynamic discretisation and minimum description length (Fayyad and Irani, 1993) might improve the BN quality. It is generally hard to specify a discretisation method a priori, as different methods work well for different applications; however, it would be interesting if the discretisation process is not separated and independent of the structure learning process. An open question is whether BN learning would improve if the discretisation and structure-learning processes were informed by each other. We hypothesise that the structure-learning processes would benefit from discretising the data in a way that gives the best goodness of fit. Furthermore, the MPC-HC algorithm comprises two components: the skeleton estimation and the edge orientation. These two parts are independent; therefore, one can use them separately or in combination with other algorithms. For example, one can estimate the skeleton using partial correlations, the Max–Min Parents and Children algorithm (Tsamardinos et al., 2006), or a skeleton constructed from expert knowledge, then orient the edges as detailed above.

Once a BN is estimated, a joint probability distribution is established over the variables. There are many applications for a joint probability distribution. Besides identifying the conditional independence of the variables, a joint probability distribution can be used to make predictions, perform sensitivity analysis or as a generative model to sample new data instances. This could alleviate the problem of inadequate training of machine learning algorithms that necessitate large sample sizes. Moreover, this work investigates the potential of BNs with a set of metrics to conduct model sensitivity analysis, assess prediction accuracy, and evaluate the uncertainty of the predictions with PPCI. It is worth mentioning that calibration measures are not explicitly included in the analysis because BNs are well-calibrated models.

In this work, the markers are measured at a single time point; future work will extend the proposed framework to investigate the preterm phenotype over a spectrum of gestational ages to investigate the temporal evolution of markers of interest, including measurements from other imaging modalities and behavioural outcomes. Moreover, the presented framework could be applied to analyse other complex brain disorders, such as attention deficit hyperactivity and autism spectrum disorders and how such disorders compare to preterm birth. In addition, the list of markers studied here might be limited and restricted to a few measurements from three different sources; however, it provides a good representation of the neuroimaging and psychological features usually studied in preterm birth. Therefore, more extensive and different markers could be employed.

## 5 Conclusions

In this study, we propose BNs as a tool to analyse heterogeneous data and present a novel structural learning algorithm (MPC-HC). We show the potential of using the BNs as a multifunctional method able to perform predictions, model the relationship between variables, investigate the effect variables have on each other, and model confidence. Furthermore, we apply BN analysis to a dataset of FT and EP subjects to investigate the effect of preterm birth on psychological and neuroimaging measurements; the findings yield results consistent with the common understanding of preterm birth. Although many questions about the effects of preterm birth remain unanswered, the preterm birth research community, other areas of brain development, and biomarker detection research might benefit from techniques like those developed by this work.

## Supplementary Material

Figure 1

## Figures and Tables

**Table 1:** The states of a node (variable) and the cut-off values for discretising each psychological and neuroimaging variable. The pink, grey, and yellow variables indicate the psychological, axonal density, and fMRI variables, respectively.

States	Variables/Markers											
	VWM	VSM	VCI	PRI		PRF	DGM	SMA		PDN	SPN	DSN
Low	<99.4	<91.7	<92.1	<91.2		<41.4	<21.8	<18.3		<0.61	<0.61	<0.63
High	>= 99.4	>= 91.7	>= 92.1	>= 91.2		>= 41.4	>= 21.8	>= 18.3		>= 0.61	>= 0.61	>= 0.63

## Data Availability

The authors do not have permission to share data.
