# Supplementary material for: Multi-source multi-modal markers for Bayesian Networks: Application to the extremely preterm born brain
Source: Med Image Anal. Author manuscript; Available in PMC 2024 Jul 12. (PMC7616207; doi:10.1016/j.media.2023.103037)
Supplement: Figure 1 [file EMS197103-supplement-Figure_1.pdf]

Multi-source multi-modal markers for Bayesian Networks: application to the extremely preterm born brain (supplementary material)

Hassna Irzan[a,b,*], Michael Hütel[a], Helen O'Reilly[d,c], Sebastien Ourselin[a], Neil Marlow[c], Andrew Melbourne[a]

[a] *School of Biomedical Engineering & Imaging Sciences, King's College London, UK*
[b] *Department of Medical Physics and Biomedical Engineering, University College London, UK*
[c] *Institute for Women's Health, University College London, London, UK*
[d] *Department of Psychology, University College Dublin, Dublin, Ireland*

1. Definition of the brain areas

The prefrontal cortex (PRF) is given by combining the anterior orbital gyrus, frontal pole, gyrus rectus, lateral orbital gyrus, medial frontal cortex, middle frontal gyrus, medial orbital gyrus, the medial segment of the superior frontal gyrus, orbital part of the inferior frontal gyrus, and superior frontal gyrus. The sensory-motor areas (SMA) are obtained by merging the medial segment of the precentral and postcentral gyri, postcentral gyrus, precentral gyrus, and supplementary motor cortex. Finally, the deep grey matter regions (DGM) are the amygdala, caudate, hippocampus, putamen, thalamus, and pallidum. Figure 1 shows a graphical representation of these areas.

*Corresponding author
Email address: hassna.irzan@kcl.ac.uk (Hassna Irzan)

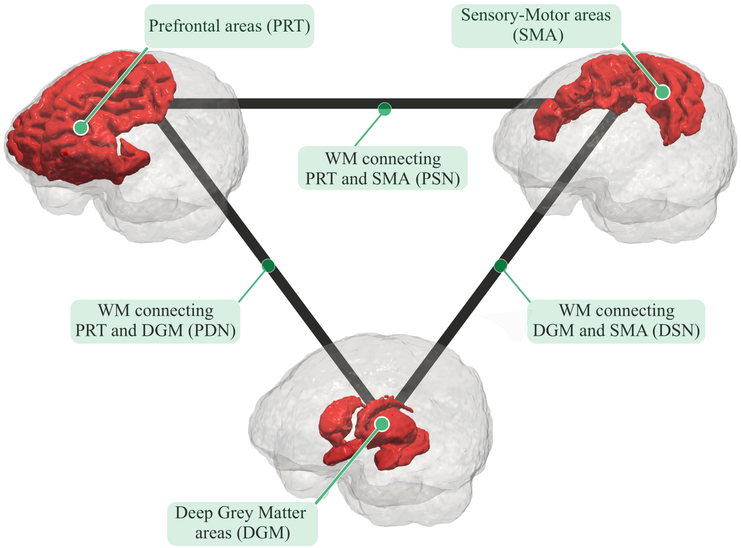

Figure 1: The brain areas in red are the grey matter areas (ROIs), while the black lines represent the white matter bundles between these areas. In the analysis, we estimate functional connectivity of the grey matter areas (ROIs) and neural density of the white matter bundles; these are given by the edge weights of the functional and microstructural networks F_n and S_n, respectively.

2. Effect of statistical threshold

We examine how varying the statistical threshold α impacts the BN structure learning with the MPC-HC algorithm. Specifically, we estimate the structure of benchmark BNs using the MPC-HC algorithm with a sample size of $M = 3000$ and two different thresholds: a low threshold of $\alpha = 0.001$ and a high threshold of $\alpha = 0.5$. Figure 2 illustrates that when employing a low threshold ($\alpha = 0.001$), the algorithm fails to capture all the edges of the skeleton, resulting in the absence of true edges in the final structure. Nevertheless, the existing edges are correctly oriented. In contrast, Figure 3 reveals that a high threshold ($\alpha = 0.05$) leads to retrieving both true and spurious edges in the skeleton. Still, the algorithm effectively eliminates excessive edges and restores the accurate structure. Consequently, the MPC-HC algorithm can successfully recover the true BN structures and eliminate erroneous edges. Furthermore, even in cases

where the skeleton lacks true edges, the algorithm orients the available edges.

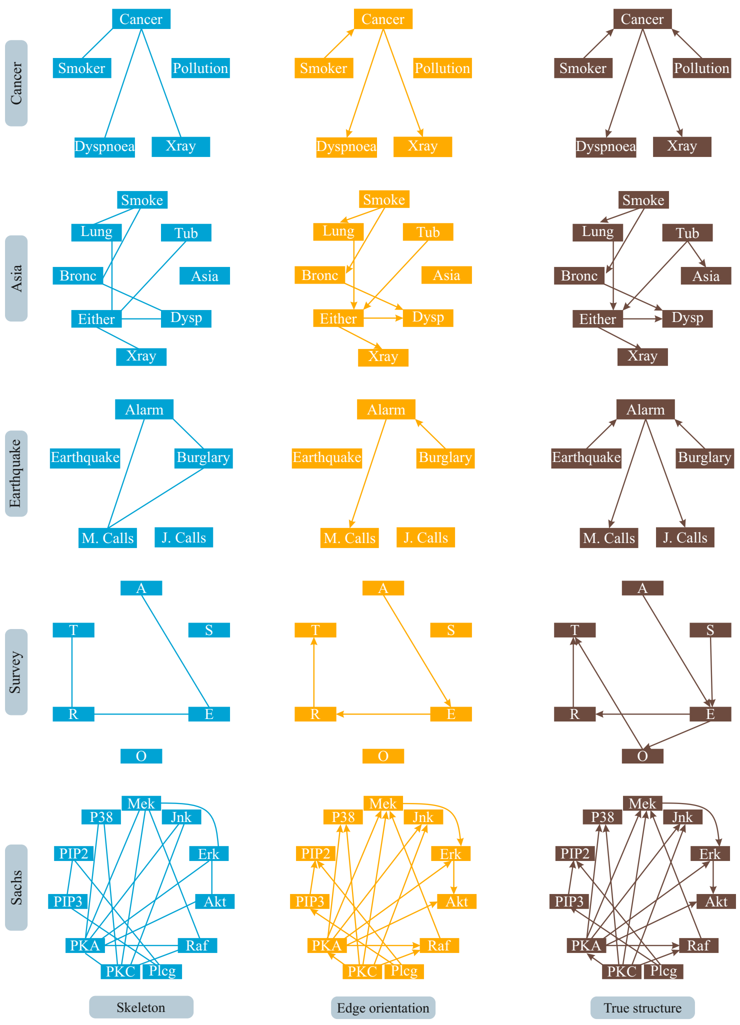

Figure 2: Structural estimates for benchmark Bayesian Networks (BNs) using the MPC-HC Algorithm with $\alpha = 0.001$. Blue outlines the network skeletons, orange lines indicate their orientation and the true structures are highlighted in brown for reference.

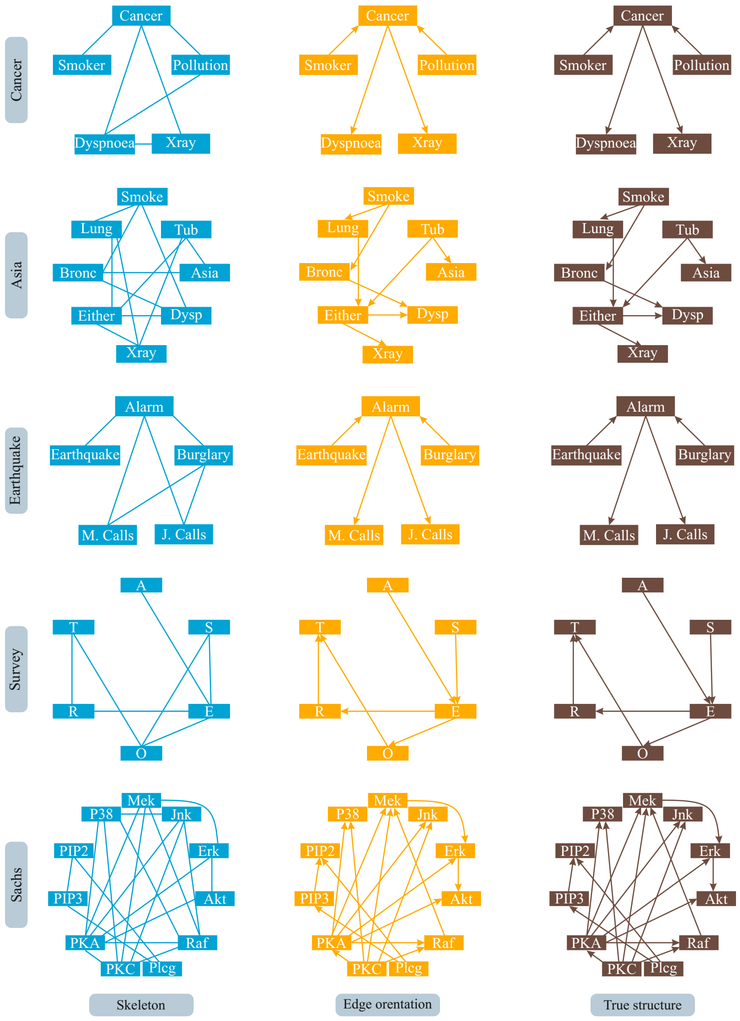

Figure 3: Structure estimates of benchmark Bayesian Networks (BNs) using the MPC-HC algorithm with a statistical threshold of $\alpha = 0.5$. The skeletons are in blue, and the oriented structures are in orange. For reference, the true structures are shown in brown.

3. Effect of the sample size

Our study investigates the impact of varying sample size (M) on the ability to recover the true structure of benchmark Bayesian Networks (BNs). We use the benchmark BNs because the ground truth structures are known. The experiment estimates the structure of benchmark BN structures using four different

sample sizes from high to low (M=5000, 2000, 1000, 100). The experiment assesses confidence in edge estimation through bootstrapping. This is done by performing the analysis presented in Section 2.3.4 of the main manuscript. Specifically, we estimate the confidence in estimating the BN edges. The results are illustrated in Figure 4. We present the results utilising the adjacency matrix of each BN. The figure shows that the confidence of the MPC-HC algorithm increases with a larger sample size.

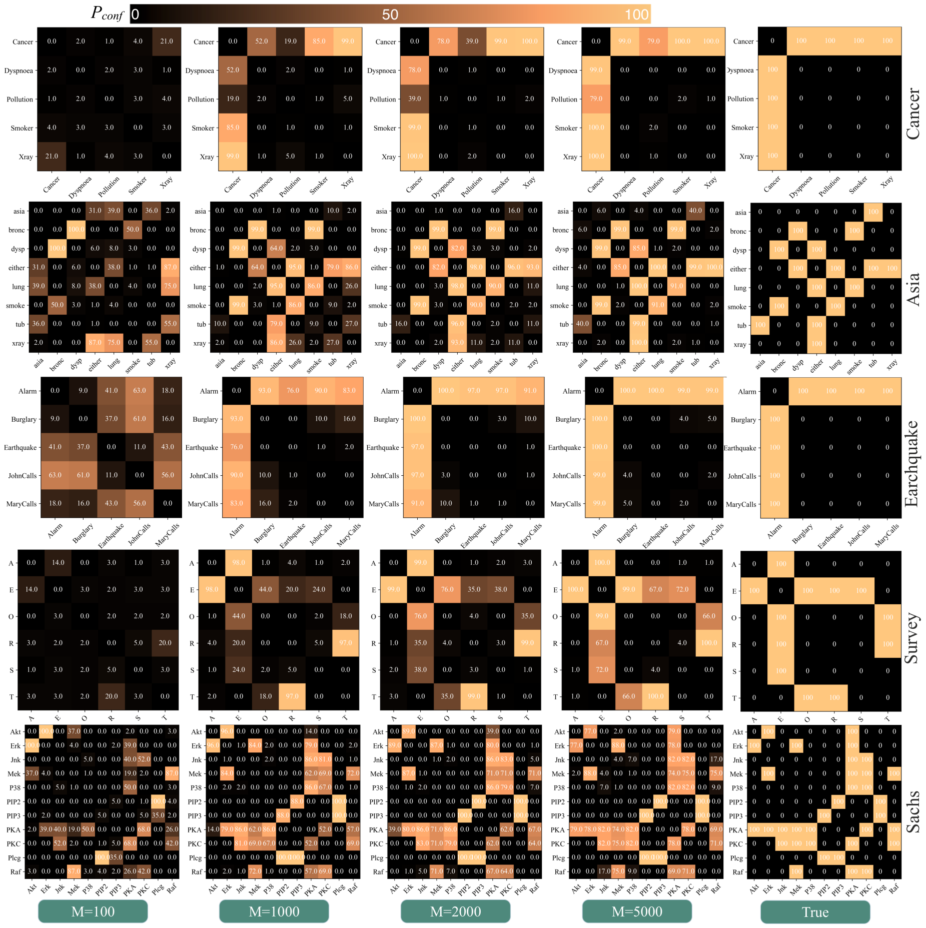

Figure 4: Heatmap of the confidence that an edge is part of the BN. The confidence is estimated using different sample sizes from $M = 100$ to $M = 5000$. The values inside each entry of the adjacency matrices are the frequency by which each edge has been estimated over 100 iterations. On the right, the adjacency matrix of the true edges is also shown.

4. Complexity of MPC-HC

The number of conditional independence required is bounded by an upper maximum of:

$$2\binom{O}{2}\sum_{i=0}^{b}\binom{O-1}{i}$$

where O is the number of nodes and b the maximal degree of any node; however, this upper bound is rarely reached. From our empirical evaluation, the time complexity of conditional independence tests is negligible with respect to edges orientation. For the edge orientation step, we analyse the size of the search space by examining the worst-case scenario case. In general, given a set of immoralities of size r, there are t-combinations of this set, with t ranging from one to r. Hence:

$$\sum_{t=1}^{r}\frac{r!}{t!(r-t)!} = 2^r - 1 \tag{1}$$

There are $2^r - 1$ graphs produced by the combinations of immoralities, to that we add one to account for the graph that has no immoralities, therefore there are 2^r graphs to be searched. Empirically, this limit is an upper bound as the search space is smaller because some immoralities cannot be combined as they would produce cyclic graphs.

5. Structural Hamming Distance (SHD) and F1 Scores

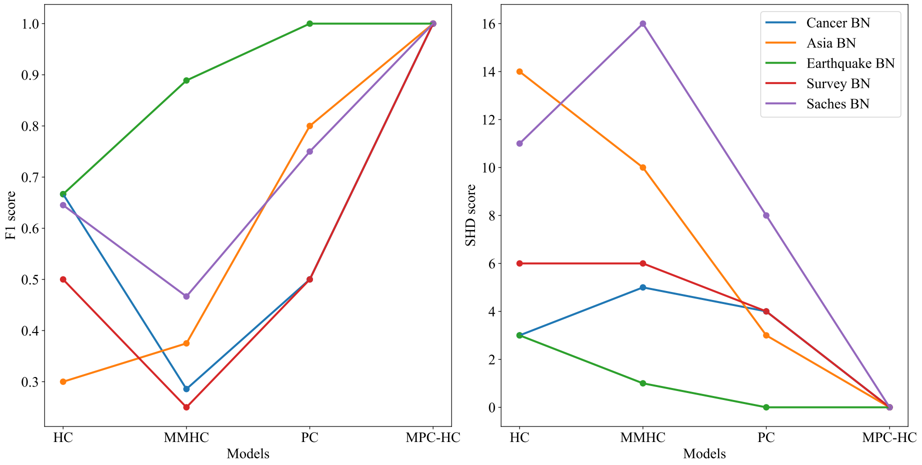

Figure 5: Comparison of F1 and Structural Hamming Distance (SHD) scores between the HC, MMHC, PC, and MPC-HC models. The MPC-HC model achieves the optimal F1 (one) and SHD (zero) scores across all the benchmark BNs. Following is PC, with HC ranking third and MMHC performing last in this evaluation.
